# Prevalence of and risk factors for enlarged perivascular spaces in adult patients with moyamoya disease

**DOI:** 10.1186/s12883-017-0935-x

**Published:** 2017-08-04

**Authors:** Tomoyoshi Kuribara, Takeshi Mikami, Katsuya Komatsu, Hime Suzuki, Hirofumi Ohnishi, Kiyohiro Houkin, Nobuhiro Mikuni

**Affiliations:** 1 Department of Neurosurgery, Sapporo Medical University, South1 West16, Chuo-ku, Sapporo, 060-8543 Japan; 2 Department of Public Health, Sapporo Medical University, Sapporo, Japan; 3 Department of Neurosurgery, Hokkaido University Graduate School of Medicine, Sapporo, Japan

**Keywords:** Moyamoya disease, Small vessel disease, Leukoaraiosis, Hypertension

## Abstract

**Background:**

Enlarged perivascular spaces (EPVS) are often observed with magnetic resonance imaging in patients with small vessel disease. However, the risk factors, radiological features, and clinical relevance of EPVS in patients with moyamoya disease are poorly understood. The purpose of this study was to evaluate EPVS, the risk factors of many EPVS, and the pathophysiology of EPVS in adult patients with moyamoya disease.

**Methods:**

One hundred cerebral hemispheres of 50 adult patients with moyamoya disease were examined. The control group consisted of 50 age/sex-matched patients without ischemic disease. The numbers of EPVS at the level of the centrum semiovale per hemisphere were compared between the moyamoya disease and control groups. In each hemisphere, the total numbers of EPVS were categorized into five grades (0–4), and the clinical and radiological characteristics of the predictive factors in patients in the high EPVS grade group (EPVS grade = 4) were assessed.

**Results:**

The EPVS counts and grades were significantly higher in the moyamoya disease group. Analyses of the background characteristics of the patients with moyamoya disease revealed that significantly higher prevalence of high EPVS grades were associated with the female sex, hypertension, high magnetic resonance angiography scores, high numbers of flow voids in the basal ganglia, high brain atrophy scores, ivy signs, and white matter lesions. A logistic multivariate analysis of the patients with high EPVS grades revealed significant associations with the female sex, hypertension, and flow voids in the basal ganglia.

**Conclusions:**

Increased EPVS were confirmed in adult patients with moyamoya disease, and the associated clinical and radiological factors were identified. The presence of hypertension, the female sex, and flow voids in the basal ganglia were important for predicting high EPVS grades in patients with moyamoya disease. Reductions in arterial pulsations with steno-occlusive changes can inhibit the flow of interstitial fluid, which can increase the number of EPVS in patients with moyamoya disease. Other clinical factors, such as the female sex and hypertension, may promote secondary brain damage in patients with moyamoya disease. Further evaluations of EPVS in patients with moyamoya disease are needed to better understand their pathophysiological importance.

## Background

Moyamoya disease is a cerebrovascular disease that is characterized by chronic progressive stenosis of the terminal portion of the internal carotid artery (ICA) on both sides of the brain, which results in an abnormal vascular network of the collateral pathways at the base of the brain [1, 2]. Therefore, patients with moyamoya disease have decreased cerebral blood flow and reduced cerebral perfusion pressure [3], which can result in ischemic and/or hemorrhagic strokes. In patients with chronic hypoperfusion, ischemic injury to the white matter causes axonal destruction and glial proliferation [4]. As a result, white matter lesions are observed in these patients on T2-weighted magnetic resonance imaging (MRI) and fluid-attenuated inversion recovery (FLAIR) imaging [5]. Similarly, hemodynamic stress can induce microbleeds [6]. Thus, the deep white matter imaging findings in patients with moyamoya disease resemble those associated with patients with small vessel disease [7].

Perivascular spaces, which are also known as Virchow-Robin spaces, surround the walls of perforating arterioles and venules as they course from the subarachnoid space through the brain parenchyma [8]. Enlarged perivascular spaces (EPVS) are commonly detected on T2-weighted MRI as dot-like or linear hyperintensities in the basal ganglia and the centrum semiovale, and this is thought to be one of the radiological features of small vessel disease [9, 10]. EPVS are more numerous in patients with greater age [11–13], hypertension [11, 13, 14], vascular dementia [15], lacunar stroke [10, 11], and white matter lesions [10–12], and EPVS are considered markers of small vessel disease. Although EPVS have been observed in patients with moyamoya disease, their characteristics in patients with moyamoya disease have never been described. In this study, EPVS were compared between patients with moyamoya disease and those in the control group, and the risk factors for increased numbers of EPVS in moyamoya disease were identified.

## Methods

### Patients

Between August 2008 and April 2016, consecutive patients with moyamoya disease who were diagnosed at our hospital were enrolled in this study. The patients were diagnosed with moyamoya disease with MRI, magnetic resonance angiography (MRA), and/or digital subtraction angiography. Patients under 15 were excluded. One hundred 100 hemispheres of 50 patients (14 males and 36 females) with moyamoya disease were examined. The characteristics of the patients are presented in Table 1. The median age (interquartile range: IQR) of the patients was 41.5 (31.0-51.0) years (range, 20–74 years). In this moyamoya disease group, 18 hypertension patients, 1 type 2 diabetes mellitus patient, and 8 hyperlipidemia patients were included. Of these 50 individuals, 25 had ischemic onsets, 6 had hemorrhagic onsets, 2 had epileptic onsets, and the remaining 17 were asymptomatic or reported only headaches. The median MRA score was 4.0 (3.0-7.0) (range, 0–10). Five patients with unilateral involvement were included in the study. Because patients with quasi-moyamoya disease were excluded from the study, none of the patients had any concomitant diseases. For the control group, 50 age- and sex-matched patients (14 males and 36 females) who were treated at our hospital during the same period were recruited. The control group included 17 patients with cerebral aneurysms, 4 migraine patients, 2 patients with cerebral hemorrhages, 5 patients with brain arteriovenous malformations, 5 patients with epilepsy, 7 brain tumor patients, and 10 patients with other issues. The patients in the control group did not have ischemic cerebrovascular disease. Therefore, these were without TIA or previous ischemic stroke, and without intracranial and extracranial stenotic lesions. The median age of the control group was 40.5 (33.8-50.3) years (range, 15–69 years). In this control group, 9 hypertension patients, 1 type 2 diabetes mellitus patient, and 7 hyperlipidemia patients were included. Compared with the moyamoya disease group, the clinical characteristics of the control group were not significantly different except for hypertension.

**Table 1:** Clinical characteristics of the moyamoya disease and control groups

	Moyamoya disease group	Control group	*p* value
n, patients (hemisphere)	50 (100)	50 (100)	
Age, median (IQR), y	41.5 (31.0-51.0)	40.5 (33.8-50.3)	0.629
Sex, male/female	14/36	14/36	1.000
Total number of EPVS, median (IQR)	43.8 (26.0-52.9)	23.8 (15.1-33.0)	<0.001
EPVS Grade, median (IQR)	4.0 (3.0-4.0)	3.0 (2.0-3.0)	<0.001
Grade 0/1/2/3/4, n	0/2/12/33/53	0/8/30/44/18	
Interobserver discrepancy, n, median (IQR)	13, 0.0 (0.0-0.0)	25, 0.0 (0.0-0.8)	
Hypertension, presence/absence	18/32	9/41	0.043
Diabetes mellitus, presence/absence	1/49	1/49	1.000
Hyperlipidemia, presence/absence	8/42	7/43	0.779
Stroke lesions (total), presence/absence	32/68	6/94	<0.001
Lacnar stroke, presence/absence	15/84	4/96	0.008
Large ischemic stroke, presence/absence	13/88	0/100	<0.001
Hemorrhagic stroke, presence/absence	4/96	2/98	0.683
Microbleeds, presence/absence	15/83	4/96	0.007
White matter lesions, presence/absence	57/43	34/66	0.001
Disease subtype	Ischemic stroke: 10	Cerebral aneurysm: 17	
	TIA: 15	Migraine: 4	
	Hemorrhagic stroke: 6	Cerebral hemorrhage: 2	
	Epilepsy: 2	Brain AVM: 5	
	Other: 17	Epilepsy: 5	
		Brain tumor: 7	
		Other: 10	

*IQR* interquartile range, *TIA* transient ischemic attack, *AVM* arteriovenous malformation

In this study, the numbers and grades of EPVS were compared between the moyamoya disease and control groups. Interobserver discrepancies were also assessed. The 100 hemispheres that were analyzed in this study were divided into the following two groups according to their EPVS grades: high EPVS grade group (EPVS grade = 4) and low EPVS grade group (EPVS grade = 0–3). First, the following clinical and radiological characteristics were compared between the moyamoya disease group and the control group for assessing selection bias: age, sex, hypertension, diabetes, hyperlipidemia, disease subtype, presence or absence of stroke lesions, microbleeds, and white matter lesions. Stroke lesions were defined as follows: lacnar stroke (maximum diameter ≤ 1.5 cm), large ischemic stroke (maximum diameter > 1.5 cm), and hemorrhagic stroke. Next, the following clinical and radiological characteristics were compared between the high EPVS grade group and the low EPVS grade group in the moyamoya disease group for assessing risk factors of high EPVS in moyamoya disease: age, sex, hypertension, diabetes, hyperlipidemia, disease subtype, MRA score, number of flow voids in the basal ganglia, brain atrophy score, presence or absence of stroke lesions (lacnar stroke, large ischemic stroke, and hemorrhagic stroke), ivy signs, microbleeds, and white matter lesions.

### MRI and MRA examinations

The MRI examinations, including T2-weighted MRI, FLAIR MRI, T2*-weighted MRI, and three-dimensional time-of-flight spoiled gradient-recalled echo sequence (3D TOF SPGR), were performed with a 3.0-T magnetic resonance system (Signa Excite, Ver. 11; GE Healthcare, Milwaukee, WI, USA) The imaging parameters were as previously described [5, 16]. Volume rendering and maximum intensity projection were applied as postprocessing techniques to aid in the evaluation.

**Fig. 1:**
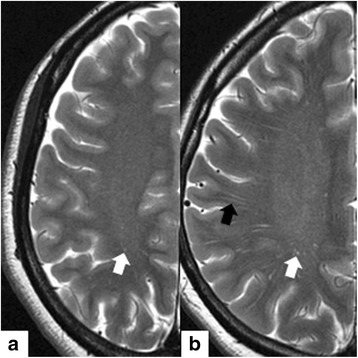
This figure shows hemispheres from a patient in the low EPVS group (**a**) and a patient in the high EPVS group (**b**). In the left hemisphere of the patient with a low EPVS grade (a 38-year-old female), the mean number of EPVS was 10.5, and the hemisphere was categorized as a grade 1 (**a**). In the left hemisphere of the patient with a high EPVS grade (a 36-year-old female), the mean number of EPVS was 83, and the hemisphere was categorized as a grade 4 (**b**). The white arrow shows dot-like EPVS, and the black arrow shows linear EPVS

All MRI and MRA data were independently evaluated in each hemisphere of each subject by two independent observers (T.K. and T.M.), and the mean of the observers’ values was calculated for the assessment. EPVS was measured on presurgical T2-weighted imaging at the level of the centrum semiovale as shown in Fig. 1. EPVS were defined as <3 mm round or linear cerebrospinal fluid-isointense lesions (hyperintense on T2-weighted and hypointense on FLAIR imaging with respect to brain) along the courses of penetrating arteries [9, 10]. Hemispheres were scored according to the mean number of EPVS as follows: 0 = none, 1 = 1–10, 2 = 11–20, 3 = 21–40, and 4 = > 40 EPVS per side [17]. In this study, EPVS in the basal ganglia could not be evaluated because of the characteristic flow voids in the basal ganglia in moyamoya disease. The number of flow voids in the basal ganglia was determined with three-dimensional time-of-flight spoiled gradient-recalled echo sequence imaging of a slice of the basal ganglia as previously described [18]. The brain atrophy scores were assessed in deep lesions (enlargement of the ventricles) and superficial lesions (enlargement of the gyri) and rated on a validated scale of 0 to 3 (normal, slight, moderate, and severe) against a reference MRI brain template of normal subjects [19, 20]. The MRA scores were based on Houkin et al.’s classification [21]. Depending on the severity of the steno-occlusive changes, MRA scores were assigned to each of the following: C1 portion of the ICA, M2 portion of the middle cerebral artery (MCA), A1 portion of the anterior cerebral artery (ACA), and P2 portion of the posterior cerebral artery (PCA). The smallest score was 0, and the largest was 10 (ICA3 + MCA3 + ACA2 + PCA2 = 10). The total score was calculated for each hemisphere. The presence of stroke lesions, ivy signs, and white matter lesions was evaluated with T2-weighted and FLAIR imaging, and the presence of microbleeds was determined with T2*-weighted imaging.

### Statistical analysis

The data are expressed as median (IQR). Mann–Whitney U tests and Chi-squared test were used to compare the moyamoya and control groups and the high EPVS grade and low EPVS grade groups. For tests that resulted in *p* values less than 0.10 in the Mann–Whitney U tests and Chi-squared test, a simple logistic regression was used in the univariate analyses of the EPVS in the moyamoya group. Odds ratios (ORs) and 95% confidence intervals (CIs) were obtained with these models. Each item was selected with stepwise methods (model selection criterion: α = 0.10), and multivariate analyses were performed on all potential predictive factors that were associated with EPVS in the univariate analysis. The statistical analyses were performed with SPSS software (version 22; IBM Corporation, Armonk, NY, USA). *p* values less than 0.05 indicated statistical significance.

## Results

The characteristics of the patients comparing the moyamoya disease group and the control group are presented in Table 1. The median ages and the percentage of females did not significantly differ, meaning that the median ages and sex tended to be similar in the groups. The median number of EPVS was significantly increased (*p* < 0.001) in the moyamoya group (43.8 [26.0-52.9]) compared with the control group (23.8 [15.1-33.0]). The median EPVS grade was significantly higher (*p* < 0.001) in the moyamoya group (4.0 [3.0-4.0]) compared with the control group (3.0 [2.0-3.0]). The distribution of the EPVS grades was as follows: grade 0 = 0, grade 1 = 2, grade 2 = 12, grade 3 = 33, and grade 4 = 53 in the moyamoya group and grade 0 = 0, grade 1 = 8, grade 2 = 30, grade 3 = 44, and grade 4 = 18 in the control group. The median interobserver discrepancies in the reported EPVS grades were 0.0 (0.0-0.0) in the moyamoya group and 0.0 (0.0-0.8) in the control group. Comparing the moyamoya disease group and the control group, the prevalence of hypertension was significantly higher in the high EPVS grade group (*p* = 0.043), though the prevalence of type 2 diabetes mellitus and hyperlipidemia was not significant. Presence of stroke lesions was significantly higher in the moyamoya disease group (*p* < 0.001). Of these, the presence of lacnar stroke was significantly higher in the moyamoya disease group (*p* = 0.008), and the presence of large ischemic stroke was significantly higher in the moyamoya disease group (*p* < 0.001). There was also significant difference between the moyamoya disease group and the control group in terms of the presence of microbleeds (*p* = 0.007) and the presence of white matter lesions (*p* = 0.001). Based on this data, multivariate analysis was performed to assess selection bias (Table 2). For tests that resulted in *p* values less than 0.10 in the Mann–Whitney U tests and Chi-squared test, a simple logistic regression was used. In the univariate analysis, moyamoya disease had higher EPVS grades (OR, 5.51; 95% CI, 2.87-10.58; *p* < 0.001). High EPVS grades in a particular hemisphere were associated with a higher prevalence of hypertension (OR, 2.67; 95% CI, 1.40-5.07; *p* = 0.003), lacnar stroke (OR, 2.84; 95% CI, 1.09-7.44; *p* = 0.033), large ischemic stroke (OR, 3.23; 95% CI, 1.01-10.27; *p* = 0.047), microbleeds (OR, 2.87; 95% CI, 1.10-7.51; *p* = 0.032), and white matter lesions (OR, 2.69; 95% CI, 1.48-4.89; *p* = 0.001). Two items (moyamoya disease and white matter lesions) were selected with stepwise methods, and the following significant differences were noted in the multivariate analysis: High EPVS grades in hemispheres were associated with moyamoya disease (OR, 4.84; 95% CI, 2.48-9.43; *p* < 0.001) and white matter lesions (OR, 2.01; 95% CI, 1.05-3.82; *p* = 0.034).

**Table 2:** Univariate and multivariate analysis concerning background characteristics

	Univariate analysis		Multivariate analysis	
Characteristics	Odds ratio (95% CI)	*p* value	Odds ratio (95% CI)	*p* value
Moyamoya disease	5.51 (2.87-10.58)	<0.001	4.84 (2.48-9.43)	<0.001
Hypertension	2.67 (1.40-5.07)	0.003		
Lacnar stroke	2.84 (1.09-7.44)	0.033		
Large ischemic stroke	3.23 (1.01-10.27)	0.047		
Microbleeds	2.87 (1.10-7.51)	0.032		
White matter lesions	2.69 (1.48-4.89)	0.001	2.01 (1.05-3.82)	0.034

*EPVS* enlarged perivascular spaces, *SD* standard deviation

In the moyamoya disease group, the characteristics of the patients when comparing the high and low EPVS grade groups are presented in Table 3. The group with high EPVS grades included 53 hemispheres, while the low EPVS grade group included 47 hemispheres. The median number of EPVS was significantly increased (*p* < 0.001) in the group with high EPVS grades (52.0 [46.0-70.0]) compared with the group with low EPVS grades (25.0 [18.0-31.5]). The median ages were 42.0 (32.0-51.5) years in the high EPVS grade group and 41.0 (31.0-50.0) years in the low EPVS grade group, which did not significantly differ (*p* = 0.516). Thus, the median ages tended to be similar in the groups. The percentage of females was significantly higher (*p* = 0.026) in the group with high EPVS grades (81.1%; 43/53) compared with the group with low EPVS grades (61.7%; 29/47). The prevalence of hypertension was significantly higher in the high EPVS grade group (*p* = 0.040), though the prevalence of type 2 diabetes mellitus and hyperlipidemia was not significant. The median MRA score was significantly higher (*p* = 0.021) in the high EPVS grade group (5.0 [3.5-7.0]) compared with the low EPVS grade group (3.0 [2.0-6.0]). The median number of flow voids in the basal ganglia was significantly higher (*p* < 0.001) in the group with high EPVS grades (2.0 [1.5-3.5]) compared with the group with low EPVS grades (1.0 [0.0-2.0]). The brain atrophy score was significantly higher (*p* = 0.004) in the group with high EPVS grades (4.0 [3.0-5.0]) compared with the group with low EPVS grades (2.0 [1.0-4.0]). There was also significant difference between the high EPVS group and the low EPVS group in terms of the prevalence of ivy signs (*p* = 0.005) and white matter lesions (*p* = 0.006). However, the group did not significantly differ for disease subtype or the prevalence of stroke lesions and microbleeds.

**Table 3:** Clinical characteristics of the patients with high EPVS grades on T2-imaging

	High EPVS grade (4)	Low EPVS grade (0–3)	*p* value
n, hemisphere	53	47	
Total number of EPVS, median (IQR)	52.0 (46.0-70.0)	25.0 (18.0-31.5)	<0.001
Age, median (IQR)	42.0 (32.0-51.5)	41.0 (31.0-50.0)	0.516
Sex, male/female	10/43	18/29	0.026
Hypertension, presence/absence	24/29	12/35	0.040
Diabetes mellitus, presence/absence	2/51	0/47	0.497
Hyperlipidemia, presence/absence	11/42	5/42	0.168
Disease subtype	Ischemic stroke: 29	Ischemic stroke: 23	
	Hemorrhagic stroke: 5	Hemorrhagic stroke: 7	0.677
	Other: 19	Other: 17	
MRA score, median (IQR)	5.0 (3.5-7.0)	3.0 (2.0-6.0)	0.021
Flow voids in the basal ganglia, median (IQR)	2.0 (1.5-3.5)	1.0 (0.0-2.0)	<0.001
Brain atrophy score, median (IQR)	4.0 (3.0-5.0)	2.0 (1.0-4.0)	0.004
Ivy sign, presence/absence	34/19	17/30	0.005
Stroke lesions (total), presence/absence	20/33	12/35	0.192
Lacnar stroke, presence/absence	10/43	5/42	0.250
Large ischemic stroke, presence/absence	8/45	5/42	0.508
Hemorrhagic stroke, presence/absence	2/51	2/45	1.000
Microbleeds, presence/absence	9/43	6/40	0.558
White matter lesions, presence/absence	37/16	20/27	0.006

*EPVS* enlarged perivascular spaces, *SD* standard deviation, *MRA* magnetic resonance angiography

The predictive factors that were associated with EPVS in the patients with moyamoya disease are presented in Table 4. In the univariate analysis, females had higher EPVS grades (OR, 2.67; 95% CI, 1.08-6.60; *p* = 0.034). High EPVS grades in a particular hemisphere were associated with higher prevalence of hypertension (OR, 2.41; 95% CI, 1.03-5.65; *p* = 0.042), higher MRA scores (OR, 1.22; 95% CI, 1.03-1.44; *p* = 0.020), higher numbers of flow voids in the basal ganglia (OR, 1.53; 95% CI, 1.18-1.99; *p* = 0.001), higher brain atrophy scores (OR, 1.40; 95% CI, 1.10-1.78; *p* = 0.007), ivy signs (OR, 3.16; 95% CI, 1.39-7.16; *p* = 0.006), and white matter lesions (OR, 3.12; 95% CI, 1.37-7.11; *p* = 0.007). Three items (female sex, hypertension, and flow voids in the basal ganglia) were selected with stepwise methods, and the following significant differences were noted in the multivariate analysis. High EPVS grades in hemispheres were associated with the female sex (OR, 3.03; 95% CI, 1.04-8.84; *p* = 0.042), prevalence of hypertension (OR, 3.32; 95% CI, 1.23-9.00; *p* = 0.018), and higher number of flow voids in the basal ganglia (OR, 1.51; 95% CI, 1.16-1.97; *p* = 0.003).

**Table 4:** Predictive factors of high EPVS in patients with moyamoya disease

	Univariate analysis		Multivariate analysis	
Characteristics	Odds ratio (95% CI)	*p* value	Odds ratio (95% CI)	*p* value
Female sex	2.67 (1.08-6.60)	0.034	3.03 (1.04-8.84)	0.042
Hypertension	2.41 (1.03-5.65)	0.042	3.32 (1.23-9.00)	0.018
MRA score	1.22 (1.03-1.44)	0.020		
Flow voids in the basal ganglia	1.53 (1.18-1.99)	0.001	1.51 (1.16-1.97)	0.003
Brain atrophy score	1.40 (1.10-1.78)	0.007		
Ivy sign	3.16 (1.39-7.16)	0.006		
White matter lesions	3.12 (1.37-7.11)	0.007		

*EPVS* enlarged perivascular space, *MRA* magnetic resonance angiography, *CI* confidence interval

## Discussion

### Pathophysiology of EPVS in the patients with moyamoya disease

This study confirmed that the patients with moyamoya disease tended to have higher numbers of EPVS, which are characteristic findings in patients with moyamoya disease due to chronic ischemic changes. The perivascular spaces are thought to constitute a perivascular lymphatic drainage pathway for interstitial fluid and solutes, such as amyloid-beta, from the brain parenchyma. Interstitial fluid and solutes drain along narrow basement membranes in the walls of arterioles to the lymph nodes in the neck [22, 23]. This system, which is largely separate from the cerebrospinal fluid system known as the glymphatic system [22, 23], is powered by vascular pulsations [24, 25]. When the system is inhibited by reduced pulsations, interstitial fluid and solutes, such as amyloid-beta, can accumulate. The accumulation of amyloid-beta causes Alzheimer’s disease [26, 27], and the accumulation of interstitial fluid induces EPVS [13]. In moyamoya disease, chronic progressive stenosis of the terminal portion of the bilateral ICA results in an abnormal vascular network that is composed of collateral pathways at the base of the brain [1, 2]. The vessels that are derived from the collateral circulation are expected to have weak arterial pulsations compared with normal circulation, which can reduce the arterial pulsations and thereby inhibit the glymphatic system and increased EPVS counts in patients with moyamoya disease.

### Risk factors of EPVS in patients with moyamoya disease

In the multivariate analysis, hypertension, female sex, and flow voids in the basal ganglia were selected for examination because of their association with high EPVS grades in the patients with moyamoya disease.

A relationship between EPVS and hypertension has been reported in previous studies of patients with lacunar strokes and normal subjects [11, 13]. This was confirmed in the patients with moyamoya disease. Two biological mechanisms are thought to underlie the hypertension-related increase in EPVS. The first mechanism involves arteriosclerosis, which van Swieten et al. have suggested is related to EPVS [28]. Arteriosclerosis might result in a reduction in the pulsations of the arterioles and, subsequently, more EPVS. The second involves impairments in the arteriole dilatation in the brain. Patients with moyamoya disease have lower cerebral blood flow and reduced cerebral perfusion pressure due to chronic progressive steno-occlusive changes in the ICA in both hemispheres. The arterioles dilate to maintain normal cerebral perfusion, and the cerebral blood volume increases [3, 29]. As a result, the impairments in the arteriole pulsation inhibit the glymphatic system, and the subsequent accumulation of interstitial fluid results in EPVS. In other words, hypertension might promote an increase in EPVS. On the other hand, the prevalence of hypertension in the moyamoya disease group was significantly higher than that in the control group, as shown in Table 1. Moyamoya disease is often accompanied by hypertension [30, 31], and a recent study suggested that a polymorphism of RNF213 is also associated with systolic blood pressure [32]. The prevalence of hypertension is higher than that in the age-sex matched control group. Although there is a possibility that hypertension might have a statistical influence in the comparison of clinical characteristics of high EPVS grade, hypertension was not selected as a confounding factor in the multivariate analysis comparing the moyamoya disease group and the control group. Therefore, the selection bias will not be high for evaluating high EPVS.

In this study, the female sex was associated with high EPVS grades. In elderly patients and patients with cerebral autosomal-dominant arteriopathy with subcortical infarcts and leukoencephalopathy (CADASIL) syndrome, no associations between sex and EPVS counts in the central semiovale have been observed [10, 11], while the male sex has been associated with high EPVS grades in the basal ganglia [12, 13]. Our observation of a female dominancy for the EPVS was thought to be characteristic of moyamoya disease. A previous study has shown that female patients with moyamoya disease are more susceptible to the development of preoperative transient ischemic attacks and have higher risks of adverse postoperative events, despite successful revascularizations [33]. In addition, unilateral moyamoya disease is less common in female patients with moyamoya disease [33, 34], which suggests that the disease might be more aggressive in female patients, perhaps because of specific sex-related factors that affect the pathophysiology and clinical course of the moyamoya disease. Similar sex-specific differences have been described in patients with symptomatic atherosclerotic intracranial arterial stenosis [35, 36]. In those studies, sex-specific differences in coagulation and fibrinolytic pathways, which occur due to the effects of estrogen on the endothelial wall and surrounding connective tissue, X-linked genetic differences, and methylation patterns have all been suggested to explain these sex differences. Whether these or other factors that specifically influence the pathophysiology of moyamoya disease are the cause of our observed sex differences remain unclear. Because there are few cases, further observations are needed before conclusions can be made.

From the viewpoint of MRI findings, disease stage and degree of ischemia should be associated with high EPVS grade. An association between flow voids in the basal ganglia and high EPVS grades was also confirmed, even though the ORs were not strong. The development of deep parenchymal collaterals is associated with the progression of moyamoya disease [18]. The deep parenchymal collaterals and the degree of steno-occlusive changes partially affect EPVS in adult patients with moyamoya disease. Although ivy sign and white matter lesions were not selected in multivariate analysis, the odds ratio revealed high values that were significant. The ivy sign is associated with low cerebral perfusion or misery perfusion [37, 38]; therefore, high EPVS might be associated with lower cerebral blood flow. However, this is not true in pediatric cases with moyamoya disease, which can exhibit linear hyperintensities that extend into the perivascular space of the deep white matter in FLAIR imaging [39]. These linear hyperintensities, which have been called medullary streaks, are thought to be associated with vasculature [40] or might result in a stagnation of cerebrospinal fluid of the perivascular spaces [41]. Because of this difference in adult and pediatric patients with moyamoya disease, pediatric patients were not included in this study. To confirm this observation, EPVS studies that use different methods must be conducted on pediatric patients with moyamoya disease.

In terms of lacnar stroke and microbleeds, their prevalence values were higher in the high EPVS grade group than in the low EPVS grade group though it was not significant. From the viewpoint of small vessel disease, high EPVS is associated with other morphological features, such as white matter hyperintensities and lacnar stroke [8, 10, 42]. In this study, these items could not be included in the multivariate analysis because their *p* values were less than 0.10 in the Mann–Whitney U tests and Chi-squared test. Although their sample size and prevalence were small in this study, their interaction might be apparent with a greater number of cases.

### Study limitations

This study had several limitations. First, the EPVS assessments were performed with 3.0-T MRI at the level of the centrum semiovale. Therefore, the EPVS counts were relatively large, especially in the patients with high EPVS grades. Subsequently, interobserver discrepancies cannot be ruled out. In this study, two observers independently counted the EPVS and assigned each hemisphere an EPVS grade (0–4), which was undertaken in order to try to decrease the interobserver discrepancy shown in Table 2. Second, this study was a retrospective case–control study, and it did not track the progress of the EPVS in the studied hemispheres over time. The control group might not have been sufficiently rigorous in this study. Because we could not perform MRI on the normal volunteers in this study, we enrolled patients with nonischemic small lesions as controls because we considered them almost identical to normal volunteers for our purposes. The association between EPVS and the progression of the symptoms of moyamoya disease is still unclear. The pathophysiological significance of EPVS in moyamoya disease should be analyzed in future prospective cohort studies. Finally, because moyamoya disease is rare, the sample size was necessarily relatively small. Studies with larger sample sizes are needed to confirm our observations.

## Conclusions

Adult patients with moyamoya disease exhibited increased numbers of EPVS. Hypertension, the female sex, and flow voids in the basal ganglia were found to be the most important factors for predicting high EPVS grades in patients with moyamoya disease. Reductions of arterial pulsations due to steno-occlusive changes might have inhibited the flow of interstitial fluid in the patients with moyamoya disease, which would increase the number of EPVS in these patients. Other clinical factors, such as female sex and hypertension, might also promote secondary brain damage in moyamoya disease. Further evaluations of EPVS in patients with moyamoya disease are needed to better understand their pathophysiological importance.

## Abbreviations

ACA: Anterior cerebral artery
EPVS: Enlarged perivascular spaces
FLAIR: Fluid-attenuated inversion recovery
ICA: Internal carotid artery
MCA: Middle cerebral artery
MRA: Magnetic resonance angiography
MRI: Magnetic resonance imaging
PCA: Posterior cerebral artery
